# Ultrasound enhances the therapeutic potential of mesenchymal stem cells wrapped in greater omentum for aristolochic acid nephropathy

**DOI:** 10.1186/s13287-021-02243-7

**Published:** 2021-05-03

**Authors:** Yuanjun Yang, Xiaodong Geng, Kun Chi, Chao Liu, Ran Liu, Xiangmei Chen, Quan Hong, Guangyan Cai

**Affiliations:** Department of Nephrology, Chinese PLA General Hospital, Chinese PLA Institute of Nephrology, State Key Laboratory of Kidney Diseases, National Clinical Research Center of Kidney Diseases, Beijing, 100853 China

**Keywords:** Greater omentum, Mesenchymal stem cells (MSCs), Aristolochic acid nephropathy (AAN), AKI-CKD, Under ultrasound guidance

## Abstract

**Background:**

Mesenchymal stem cells (MSCs) have been reported to promote regeneration in both subjects with acute kidney injury (AKI) and chronic kidney disease (CKD), but their efficacy remains limited, probably because most of the cells accumulate in the lungs, liver, and spleen after an intravenous infusion. Therefore, ultrasound-guided administration of MSCs represents a possible approach to solve this problem. The greater omentum is used to promote cell survival due to its rich vasculature. We hypothesized that ultrasound-guided administration of MSCs combined with greater omentum might be more curative than currently available approaches.

**Methods:**

In this study, we established an aristolochic acid nephropathy (AAN) model by intraperitoneally administering aristolochic acid I sodium salt (AA-I) at a dose of 5 mg/kg body weight on alternate days for 4 weeks. Subsequently, a laparotomy was performed, and the left kidney from which the capsule had been removed was wrapped with the greater omentum. A dose of 2 × 10^7^ MSCs was injected into the space between the greater omentum and the left kidney. Equal amounts of MSCs were administered under ultrasound guidance every second week for a total of 4 treatments. Mice were sacrificed 4 weeks after surgery. Serum creatinine and blood urea levels were measured to assess renal function. qPCR, Western blot, and histological analyses were conducted to further investigate the therapeutic mechanism of MSCs.

**Results:**

Ultrasound-guided injection of MSCs into the greater omentum that surrounds the kidney enriched cells in the kidney region for up to 5 days. Renal function tests indicated that MSCs improved renal function to a great extent, as reflected by decreased blood urea nitrogen and serum creatinine levels. In addition, histological analyses showed that MSCs noticeably attenuated kidney injury, as evidenced by the amelioration of tubular necrosis and peritubular interstitial fibrosis. Mitigation of renal interstitial fibrosis was further confirmed by immunohistochemistry, qPCR, and western blotting after MSC treatment. Moreover, immunofluorescence staining revealed that MSCs alleviated inflammatory responses by increasing the counts of CD206+ cells and decreasing the counts of CD68+ cells. MSC migration was initiated in response to AA-I-treated renal epithelial cells in an in vitro migration assay.

**Conclusions:**

These findings suggested that administration of MSCs into the cavity formed by the injured kidney and the greater omentum under ultrasound guidance improved renal function, attenuated kidney injury, and mitigated renal interstitial fibrosis and inflammatory responses. Thus, this approach might be a safe and effective therapy for CKD.

**Supplementary Information:**

The online version contains supplementary material available at 10.1186/s13287-021-02243-7.

## Background

Aristolochic acids (AAs) are chemical compounds that are also known as nitrophenanthrene carboxylic acid compounds. Long-term administration of foods or traditional Chinese medicines containing AA can lead to pathological changes in the urinary system and digestive system and even organ fibrosis, urothelial malignancies, colon adenocarcinoma, invasive lobular breast carcinoma, and other conditions [1]. Researchers currently believe that the mechanism of AA-induced renal interstitial fibrosis may involve renal tubular epithelial cell (RTEC) apoptosis, renal interstitial fibroblast proliferation or activation, renal interstitial inflammatory cell infiltration, RTEC transdifferentiation, myofibroblast (MyoF) aggregation, renal small blood vessel wall ischemia, and increased production and decreased degradation of extracellular matrix (ECM), a condition that may become chronic after acute injury, i.e., acute kidney injury (AKI)-chronic kidney disease (CKD) [2].

MSCs are currently among the most widely used multipotent stem cells with the capability of proliferation and differentiation. MSCs are presumed to exert their main therapeutic effects, including inhibition of apoptosis, repair and regeneration of damaged tissue, and regulation of inflammatory responses, through secreted growth factors, cytokines, and neurotrophic factors that act in a paracrine manner [3–7]. In addition, MSCs are hypoimmunogenic and possess important immunomodulatory capabilities. Previous studies have shown that MSC transplantation has a potential to treat acute and chronic renal disease [8–14]. Moreover, the omentum flap enhances the survival of cells after transplantation via increased angiogenesis [15, 16].

The greater omentum has a rich vascular net and potential for revascularization. These beneficial characteristics of the omentum have been utilized by surgeons for many years. Covering the surface of the kidney with pedicled greater omentum after renal trauma and partial nephrectomy with complete hemostasis can reduce the occurrence of rebleeding and infection and promote healing [17]. Moreover, wrapping with greater omentum restricts the MSCs to the vicinity of the damaged kidney, thus supporting their paracrine effect. Garcia-Gomez et al. confirmed the ability of this unique omentum-mediated therapy to slow 5/6 nephrectomy-induced CKD [18]. Our previous research also confirmed that the pedicled omentum valve exerts a renoprotective effect on the kidney after 5/6 nephrectomy via anti-inflammatory and antifibrotic effects [19]. However, even if the omentum is used to prolong the survival of MSCs, their therapeutic efficacy may not be sufficient because MSCs cannot survive in the body for long periods.

Although ultrasound and magnetic resonance imaging have similar accuracy, the real-time feature of ultrasonography allows clinicians to target tissue and minimize the possible damage caused by needle shear and accidental injury. In addition, for clinicians, ultrasound is better known, with a small footprint, flexible movement, no radiation, and low costs. These advantages have made ultrasound widely accepted and used by clinicians. More importantly, ultrasound facilitates convenient MSC administration. Ultrasound has now been used in the delivery of plasmonic nanoparticle-labeled MSCs to the spinal cord [20] and the regeneration of full-thickness rotator cuff tendon tears [21].

In this study, we hypothesized that combining ultrasound-guided injection of MSCs and the advantages of the greater omentum would achieve better treatment of AAN by prolonging cell survival and increasing the frequency of cell infusion. We used red fluorescent protein-mesenchymal stem cells (RFP-MSCs) to show the survival of cells in vivo, and we constructed a mouse model of AAN followed by treatment with saline or RFP-MSCs to test this hypothesis.

## Materials and methods

### Cell culture

Bone marrow-derived RFP-labeled MSCs from C57BL/6 mice and MSC-conditioned medium were both purchased from the Cyagen Biosciences (Cyagen Biosciences, Sunnyvale, CA, USA). The cells were cultured under standard conditions (37 °C, 5% CO_2_, 90% humidity), with complete medium exchanges every other day. The supplier tested the cell surface phenotypes and trilineage differentiation potential of the cells used in the experiment. The cells were positive for the cell surface antigens CD29, CD34, CD44, and Sca-1 and negative for CD117. The certificate of analysis for the RFP-MSCs is provided in the supplemental material. The MSCs were subcultured at a density of 2.5–4.0 × 10^4^ cells/cm^2^ after they reached approximately 80–90% confluence. MSCs from the 2nd and 3rd passages were used for the experiments. All cell manipulations were performed in a biosafety hood.

### Transwell migration assay

The vertical migration of mMSCs was detected using the Transwell migration assay. Transwell inserts (6.5-mm diameter and 8-mm pore size, Corning Inc., Corning, NY, USA) were loaded with 2 × 10^4^ BM-MSCs in 200 μl of complete medium (Cyagen, Suzhou, China), and 600 μl of serum-free DMEM/F12 (Corning Inc., Corning, NY, USA) with or without 1.5 × 10^6^ cells in the lower chambers. The complete medium containing different drugs (10 μg/ml aristolochic acid I sodium salt (AA-I), Sigma, USA; 10 ng/ml TNF-α, Peprotech, USA; 5 ng/ml transforming growth factor-β1 (Tgroup). The sham control (sham+salineGF-β1), Sino Biological China) was placed in the lower well to induce cell migration. The cells were allowed to migrate at 37 °C in a humidified CO_2_ incubator for 36 h. The nonmigrated cells on the upper side of the membranes were removed using cotton swabs. Migrated cells on the lower side of the membranes were fixed with 4% paraformaldehyde for 30 min at 4 °C and stained with 0.1% crystal violet for 20 min. Five different visual fields (× 200 magnification) were randomly selected for observation and photographed under a fluorescence microscope (Olympus Optical Co., Ltd.). Quantification was performed by calculating the percentage of stained area to the total area using FIJI software.

### Experimental grouping and AA-I administration

Male C57L/B6 SPF mice (6–8 weeks old) weighing 20–25 g were purchased from Vital River Laboratories (Vital River Laboratories, Beijing, China) and were fed adaptively for 7 days in the animal center at the Chinese PLA General Hospital before the experiment. The mice were maintained in an animal facility at 19–29 °C with 12-h day/night cycles and a relative humidity of 40–70%. They were fed a standard diet of rat chow and had free access to water.

AA-I was purchased from Sigma Company. AA-I powder (20 mg) was centrifuged to ensure that all the powder moved to the bottom of the bottle, and 1 ml of aseptic PEG400 was added to prepare a storage solution with a higher concentration of AA-I (20 mg/ml). The working liquid (0.5 mg/ml) was prepared in a 15-ml centrifuge tube with an appropriate amount of PBS before injection.

**Fig. 1:**
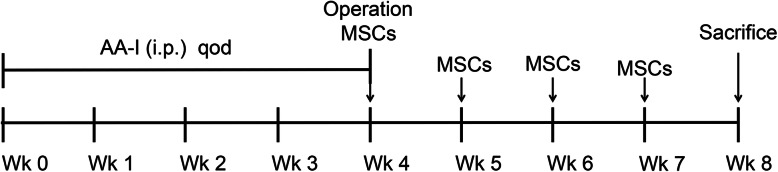
Experimental workflow of this study. The mice were randomly divided into four groups (*n* = 8/group): sham+saline, sham+MSCs, AAN + saline, and AAN + MSCs. The mice were intraperitoneally injected with saline (sham+saline, sham+MSCs) or AA-I (AAN + saline, AAN + MSCs) every other day at weeks 0, 1, 2, and 3. Starting on the day of the operation, the mice were treated with saline (sham+saline, AAN + saline) or MSCs (sham+MSCs, AAN + MSCs) weekly for a total of 4 weeks before sacrifice. i.p., intraperitoneal; qod, once every other day; wk., week

The experimental mice were divided into 4 groups (8 per group). The sham control (sham+saline) and sham-MSC (sham+MSCs) groups were intraperitoneally injected with saline every other day for 4 weeks. The AAN-control groups (AAN + saline) and AAN-MSC (AAN + MSCs) groups were intraperitoneally injected with AA-I at a dose of 5 mg/kg on alternate days for 4 weeks. A greater omentum wrapping procedure was performed at the end of the third week at the same time the first MSC or saline treatment was administered. After the operation, the mice were treated weekly with saline (sham+saline and AAN + saline) or MSCs (sham+MSCs and AAN + MSCs) under ultrasound guidance once a week for another 3 weeks. At the end of the 8th week, the mice were sacrificed. The entire experimental workflow is shown in Fig. 1.

### Cell transplantation

Under anesthesia, a left side median incision was performed after depilation of the back and routine disinfection, and the left kidney was exposed. Then, the capsule was peeled off and wrapped with free pedicled greater omentum, and 2 × 10^7^ cells (sham-MSC and AAN-MSC groups) or an equal volume of normal saline (sham-control and AAN-control groups) was injected into the cystic cavity of the greater omentum enclosing the kidney. After the injection, the wound was stitched, and the mice underwent small animal in vivo imaging using an LB983 NightOWL imaging system (Berthold Technologies). The fluorescence intensity of the kidney was determined on the day of surgery and day 5.

**Fig. 2:**
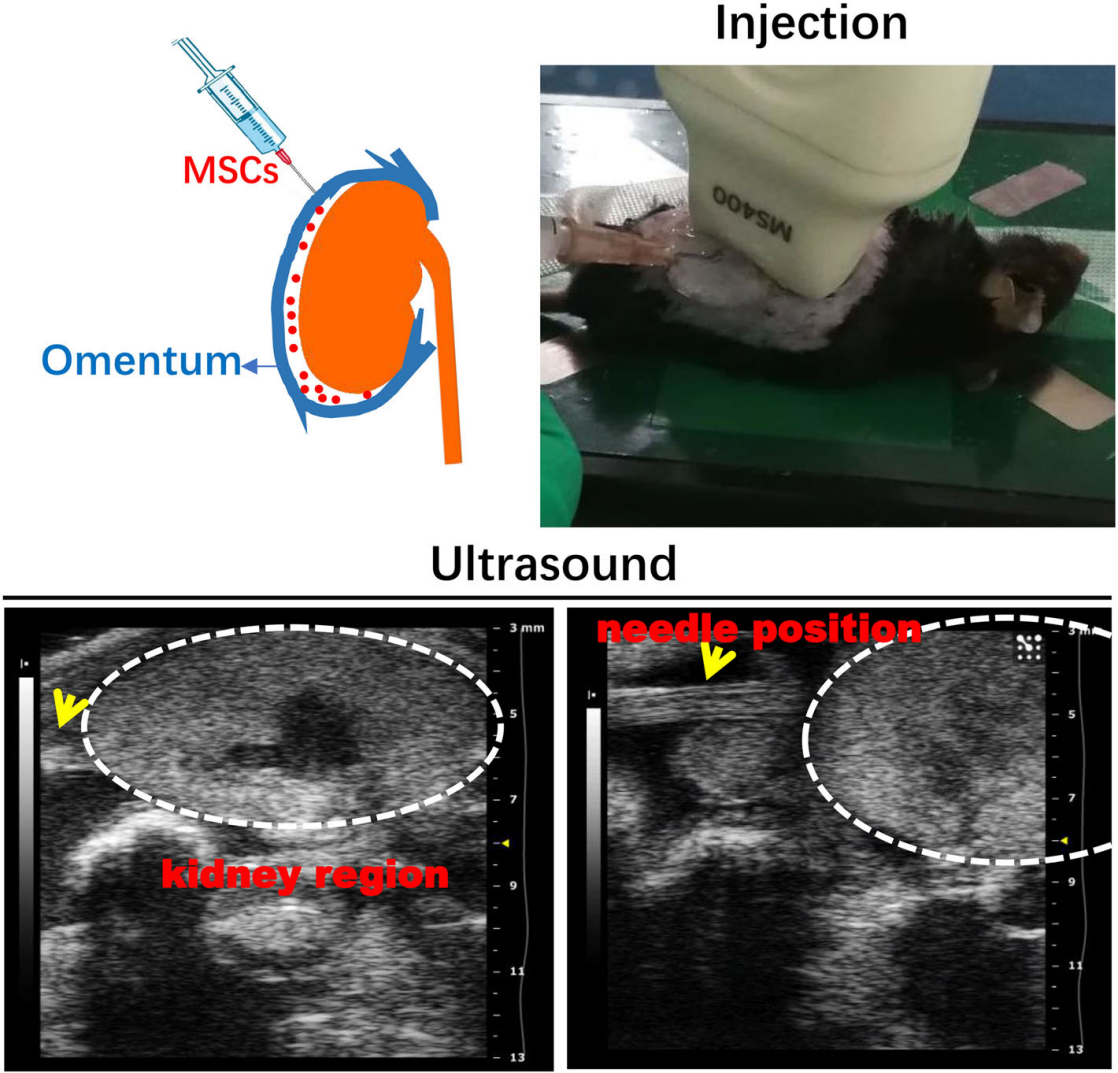
Schematic diagram depicting the treatment course of ultrasound-guided injection into the greater omentum for AAN. Ultrasound-guided MSC treatment combined with greater omentum. The oval dotted line represents the kidney region. The yellow arrow represents the needle position

After surgery, the same dose of MSCs or normal saline was injected into the cavity formed by the left kidney and the greater omentum under ultrasound guidance once a week for 4 weeks. After anesthesia, the mice were placed in a lateral position, and medical ultrasound acoustic gel (Aquasonic 100, Parker Laboratories) was placed on the left kidney region to provide acoustic coupling between the real-time microvisualization (RMV) scanhead and the skin (Fig. 2a). Under ultrasound guidance, the cells were successfully injected into the cavity formed by the injured kidney and the greater omentum using a 1-ml syringe and 26-G needle without damaging the renal parenchyma (Fig. 2b). Ultrasound imaging was performed using the Vevo 2100 system (VisualSonics, Toronto, Canada). Using B-mode imaging, the MS-400 imaging transducer (35 MHz) was positioned and held immobile to view the mouse kidney. Mice were sacrificed on the 8th weekend, and the blood and kidneys were harvested for further testing.

### In vivo fluorescence imaging

We performed both in vivo and fluorescence imaging to investigate the fate of MSCs and significant enrichment provided by the greater omentum when MSCs were administered to the animal in the location detailed in this experiment. AA-I-induced AKI was established in C57BL/6 mice described above. At the 4th weekend, mice (*n* = 3 per group) were treated with three different doses of cells (2 × 10^6^, 2 × 10^7^, and 1 × 10^8^ cells per mice) or normal saline as a control. After cell transplantation, mice were imaged in vivo using an LB983 NightOWL imaging system (Berthold Technologies) at the indicated time points (days 1, 3, 5, and 7) to track the survival of RFP-MSCs in the AA-I-induced AKI model. The fluorescence intensity of RFP-MSCs and signal to noise ratio (SNR) was measured in AAN-MSC and AAN-saline groups after the transplantation at different cell densities. The SNR was calculated using the following formula: (left kidney intensity)/(right kidney intensity).

### Two-photon fluorescence imaging of renal tissue sections

At the 5th weekend, mice were sacrificed and the kidneys were removed. The fresh kidneys were cleaned, peeled, and cut into slices with a 2-mm thickness using a sharp surgical blade. The slices were scanned with a two-photon microscope (TSP-SP5, Leica, Germany). The two-photon fluorescence emission was collected at wavelengths from 555 to 624 nm upon excitation at 543 nm for red (RFP-MSCs), and from 504 to 569 nm excitation upon excitation at 488 nm for green (autofluorescence). The autofluorescence (green) of the kidney was used as the background reference.

### Measurement of weight and renal function

The body weight of all mice was measured weekly during the study. The blood (0.2 ml) was collected once a week by cutting the tail vein to determine the serum creatinine (SCR) and blood urea nitrogen (BUN) levels. At the end of the eighth week, the mice were euthanized, and blood samples were collected from the abdominal aorta to detect SCR and BUN levels. SCR and BUN levels were measured with commercial kits (BioAssay Systems, Hayward, CA, USA).

### Real-time polymerase chain reaction (RT-PCR)

Total RNA was isolated using TRIzol (Abcam, USA), and cDNAs were synthesized using the cDNA Synthesis Kit (Applied Biosystems, Foster City, CA, USA) according to the manufacturer’s instructions. The relative expression level of the target gene was calculated using the 2^−ΔΔCT^ method. The primers used for this experiment are shown in Table 1.

**Table 1:** Primer sequences used for real-time PCR

Gene	Forward sequence (5′ → 3′)	Reverse sequence (5′ → 3′)
Mouse TNF-alpha	GGTGCCTATGTCTCAGCCTCTT	GCCATAGAACTGATGAGAGGGAG
Mouse IL-1β	TGGACCTTCCAGGATGAGGACA	GTTCATCTCGGAGCCTGTAGTG
Mouse FN	CCCTATCTCTGATACCGTTGTCC	TGCCGCAACTACTGTGATTCGG
Mouse Col-I	CCTCAGGGTATTGCTGGACAAC	CAGAAGGACCTTGTTTGCCAGG
Mouse IL-6	TACCACTTCACAAGTCGGAGGC	CTGCAAGTGCATCATCGTTGTTC

### Western blotting

Total protein was extracted from kidney tissues with RIPA lysis buffer containing protease inhibitors. The protein concentration was assayed using a BCA kit. Seventy micrograms of protein were electrophoretically separated on 10% SDS-PAGE gels and then transferred to a cellulose acetate membrane. After blocking for 1 h with a 1× casein solution, the primary antibody was added and incubated overnight at 4 °C. The mouse monoclonal anti-β-actin antibody was obtained from Sigma (St. Louis, MO, USA). Rabbit polyclonal anti-TGF-β, rabbit polyclonal anti-α-smooth muscle actin (α-SMA), and rabbit polyclonal anti-type I collagen antibodies were purchased from Abcam (Cambridge, MA, USA). After washing, the membranes were incubated with the corresponding secondary antibody. Images were analyzed using ImageJ 1.42 software.

### Histopathology

The kidney tissue was fixed with a 4% formalin solution for 24 h and then dehydrated and embedded in paraffin, cut into 3-μm sections. The left kidneys were stained with periodic acid–Schiff (PAS), Masson’s trichrome and Sirius red. The right kidneys were only stained with PAS, and not Masson’s trichrome or Sirius red. Images were acquired with a digital still camera (DP27, Olympus, Tokyo, Japan). PAS-stained sections were scored by pathologists in the Department of Nephrology who were blinded to the groups on a scale ranging from 0 to 4 points. The score indicates the extent of the renal cortical tissue that was affected by tubulointerstitial fibrosis (broadening interstitial area in between the tubules): score of 0: < 1%; score of 1: 1–5%; score of 2: 6–10%; score of 3: 11–20%; and score of 4: 21–50%. Masson’s trichrome and Sirius red staining were processed with ImageJ software for the quantitative analysis. The degree of renal fibrosis was evaluated based on the area of fibrotic staining observed in renal tissue sections at 400-fold magnification under an optical microscope. Eight mice per group and three fields of view per mouse were examined.

### Immunohistochemistry

We performed immunohistochemical staining on 3-μm kidney tissue sections using primary antibodies against α-SMA, collagen I (Col-I), phosphorylated Smad2 (p-SMAD2), CD68, and CD206 overnight at 4 °C. For α-SMA, Col-I, p-SMAD2, and CD68 staining, the sections were incubated with a biotinylated secondary antibody, subsequently treated with avidin-biotin-peroxidase complex, and finally incubated with (diaminobenzidine tetrahydrochloride) DAB peroxidase substrate solution. For the quantification of α-SMA, Col-I, p-SMAD2 staining, 3 nonoverlapping cortical sections at a magnification of 400× (HPF) were counted using Image-Pro Plus Software. 

### Immunofluorescence staining

For CD206 staining, sections were subjected to antigen retrieval, blocked in a PBS solution containing 3% BSA for 1 h, and then incubated with primary antibodies against CD206 overnight at 4 °C. Subsequently, sections were incubated with a Cy3-labeled goat anti-rabbit IgG antibody (1:300; Servicebio) for 50 min at room temperature. After 3 washes, DAPI in PBS was added and incubated for 10 min at room temperature in the dark. For the quantification of CD206 staining, 3 nonoverlapping cortical sections at a magnification of 400× (HPF) were counted using Image-Pro Plus software.

### Statistical analysis

The results are presented as the means ± standard errors of the means and were analyzed using GraphPad Prism 5.0 software (GraphPad Software, La Jolla, CA, USA) and SPSS 21.0 statistical software. Differences between multiple groups were analyzed using analysis of variance (ANOVA), whereas differences between two groups were analyzed using independent *t* tests. A *p* value less than 0.05 was declared to be statistically significant.

## Results

### AA-I-induced renal epithelial cell injury triggers MSC migration

**Fig. 3:**
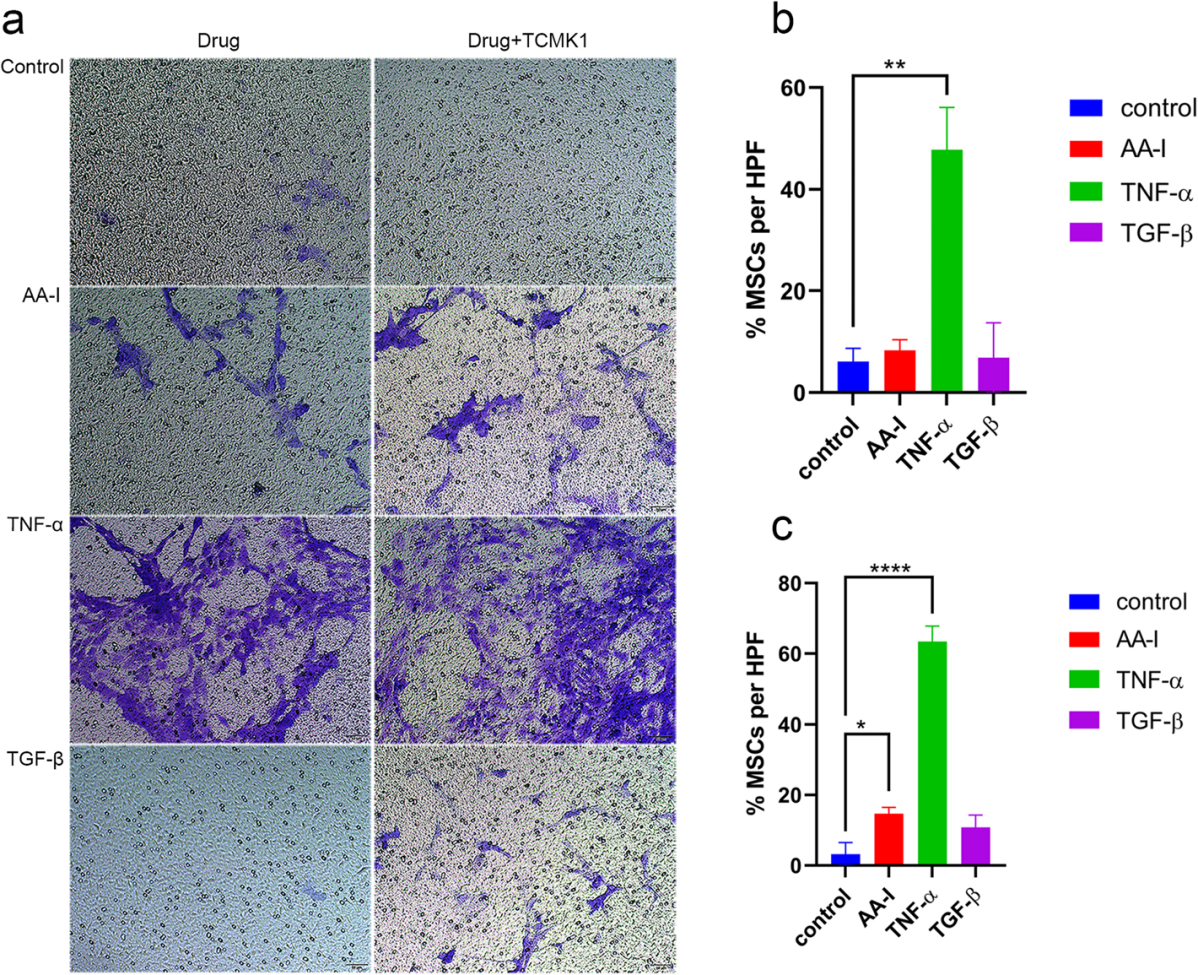
AA-I-induced renal epithelial cell injury triggers MSC migration. Inflammatory factors such as TNF-α released by injured renal epithelial cells promote AA-I-induced migration. Representative images were captured at a magnification of 200×. Scale bar = 50 μm (*P < 0.05, **P < 0.01, and ****P < 0.0001 compared with the control group)

MSCs were co-cultured with AA-I, TNF-α, or TGF-β alone or AA-I-, TNF-α-, and TGF-β-treated renal epithelial cells to examine the migratory behavior and mechanisms of MSCs exposed to AA-I. The results suggested that AA-I alone is not sufficient to cause MSC migration (Fig. 3a), but AA-I-induced renal epithelial cell injury promoted MSC migration (Fig. 3b). Furthermore, the evoked migration was most pronounced for cells exposed to the proinflammatory cytokine TNF-α with or without injured renal epithelial cells.

### Ultrasound-guided administration of MSCs combined with greater omentum effectively limits MSCs to the left kidney region at least for 5 days

**Fig. 4:**
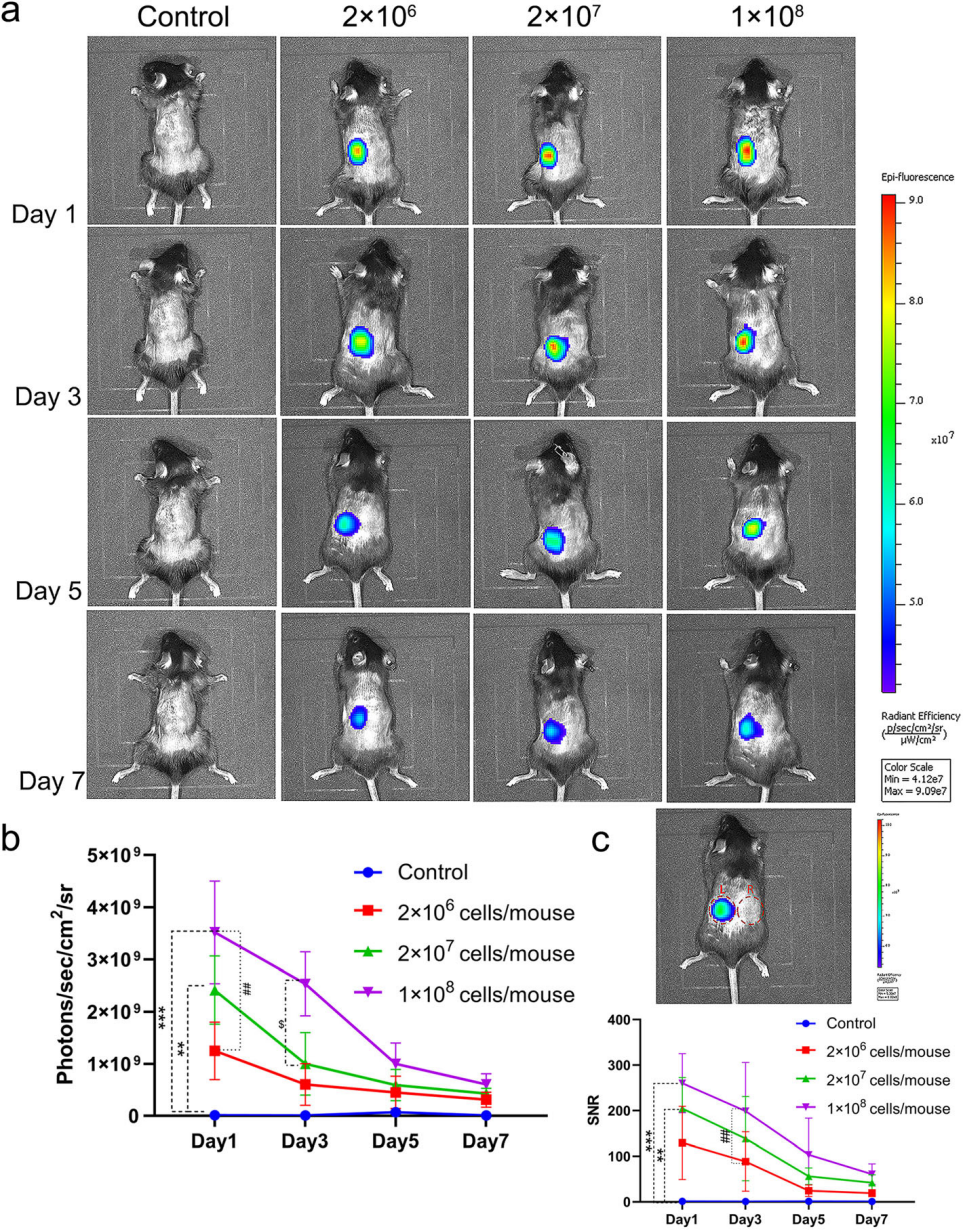
The greater omentum prolonged the survival of MSCs for at least 5 days in vivo. **a** Representative images of mice obtained using in vivo fluorescence imaging on days 1, 3, 5, and 7. The fate of RFP-MSCs after transplantation was tracked using in vivo fluorescence imaging. **b** Fluorescence intensity of RFP-MSCs after the transplantation at three different cell densities (2 × 10^6^, 2 × 10^7^, and 1 × 10^8^ cells per mice) in the AAN mouse model. **c** SNR of RFP-MSCs after the transplantation of different cell densities in the AAN mouse model. The red dashed circles represent the range of measurement allowed

As detected using small animal live imaging, the fluorescence of MSCs in the cystic cavity of the greater omentum enclosing the kidney mainly accumulated in the left kidney region, and no fluorescence was detected in the right kidney region, lung, liver, or other organs (Fig. 4a). The fluorescence intensity and SNR of MSCs gradually decreased over time and increased as the cell dose increased (Fig. 4b, c). The fluorescence intensity was substantially reduced on 5 days post MSC-transplantation. The SNR also gradually decreased over time and increased as the cell dose increased. Ultrasound-guided administration of MSCs combined with greater omentum effectively limited MSCs to the left kidney region.

**Fig. 5:**
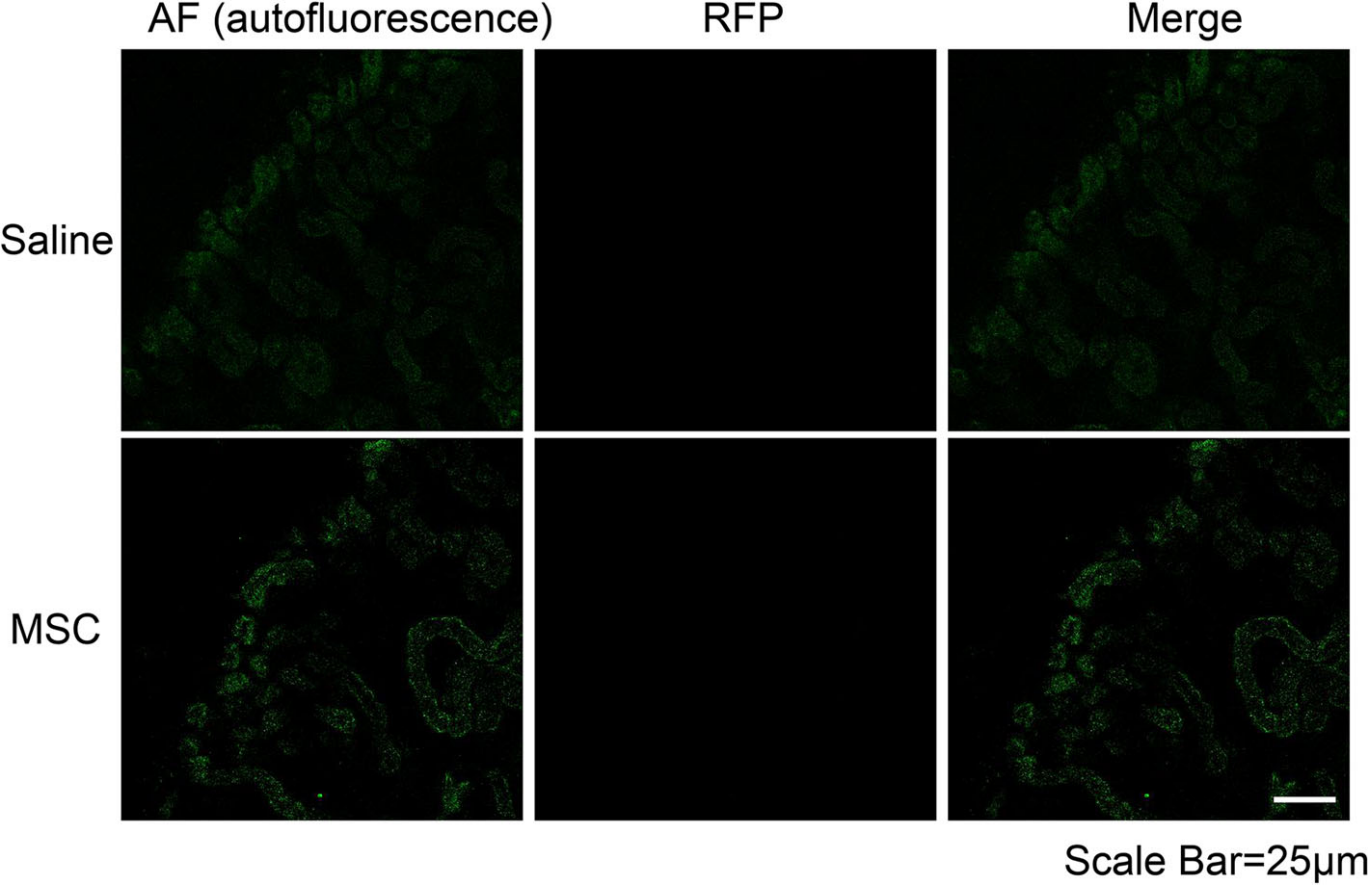
The distributions of RFP-MSCs in left kidney tissues of AAN + saline and AAN + MSC mice. MSCs did not enter the renal parenchyma, as observed using two-photon fluorescence imaging (60× oil lens; scale bar = 25 μm). The autofluorescence (green) of the kidney was used as the background reference

Two-photon laser scanning microscopy was performed to observe the left kidneys enclosed by greater omentum ex vivo and to further investigate the fate of MSCs. No red fluorescence representing MSC was observed in the left kidney tissue of the AAN-saline and AAN-MSC groups (Fig. 5). Based on these results, MSCs did not enter the renal parenchyma but may attach to the greater omentum enclosing the left kidney.

### MSCs improve damaged renal function induced by AA-I

**Fig. 6:**
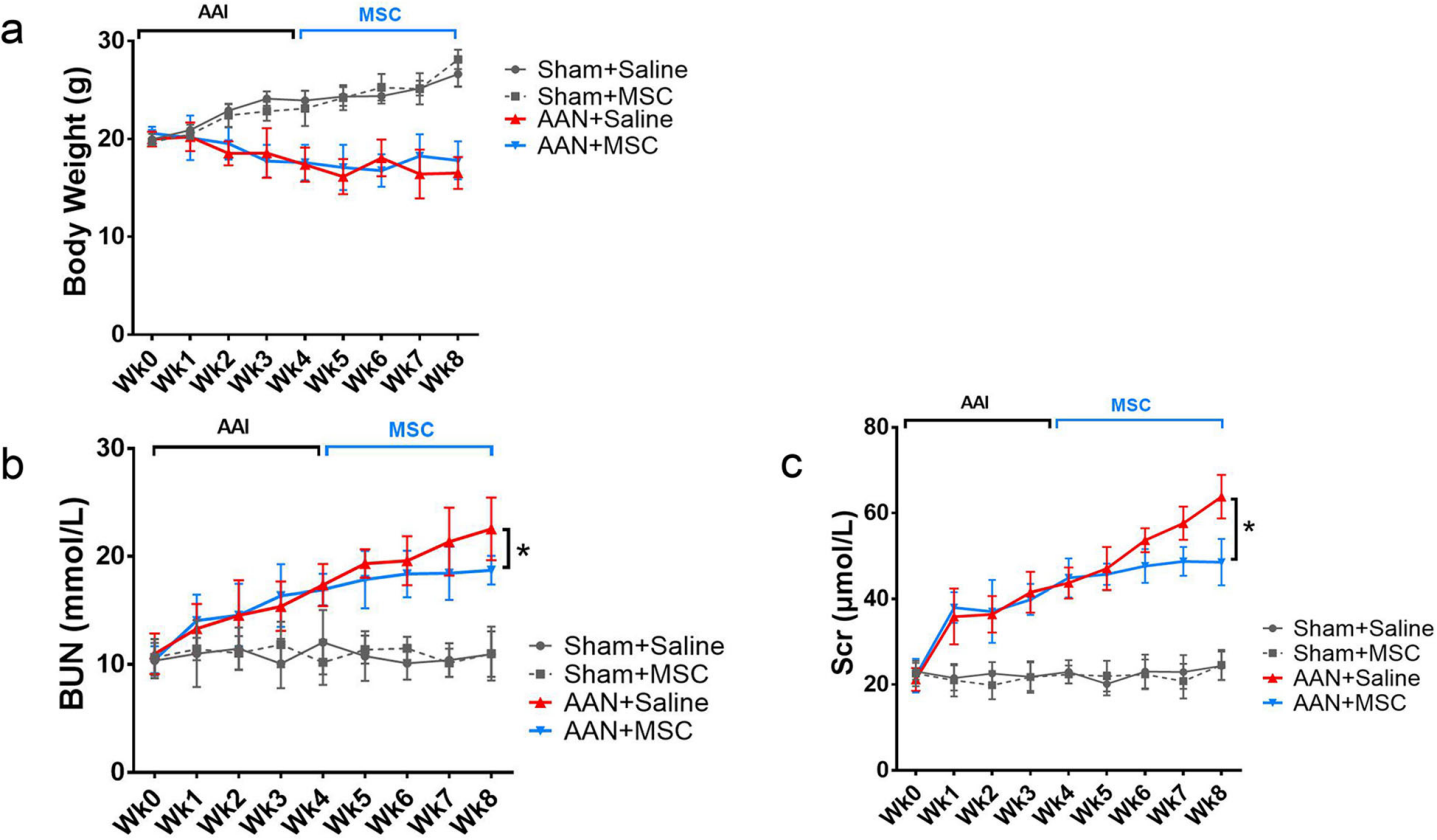
Effects of MSC-loaded greater omentum on mouse body weight and renal function indexes. Changes in body weight (**a**), BUN levels (**b**), and SCR levels (**c**) after the injection of AA-I (AAN-saline and AAN-MSCs) or saline (sham-control and sham-MSCs) every other day for 4 weeks (week 0, week 1, week 2, and week 3) and after MSC (AAN-MSCs and sham-MSCs) or saline injection for another 4 weeks (week 4, week 5, week 6, and week 7). After MSC treatment, the increased levels of SCR and BUN in the AAN-saline group were significantly reduced compared with those in the AAN-MSC group. Data are presented as means ± SEM. (*n* = 8 mice/treatment/time). *P < 0.05 for the comparison between AAN-saline and AAN-MSC mice

The body weight of mice in the sham control and sham-MSC groups (sham control and sham-MSC groups) gradually but significantly increased compared with the AAN-control and AAN-MSC groups (Fig. 6a). As expected, the AAN groups (AAN-saline and AAN-MSC groups) showed a significant increase in SCR and BUN concentrations compared with the corresponding values in the sham groups (sham control and sham-MSC groups). The increased levels of SCR and BUN observed in the AAN-saline group were significantly attenuated compared with those in the AAN-MSC group (*P* < 0.05) (Fig. 6b, c).

### MSCs ameliorate renal injury induced by AA-I

**Fig. 7:**
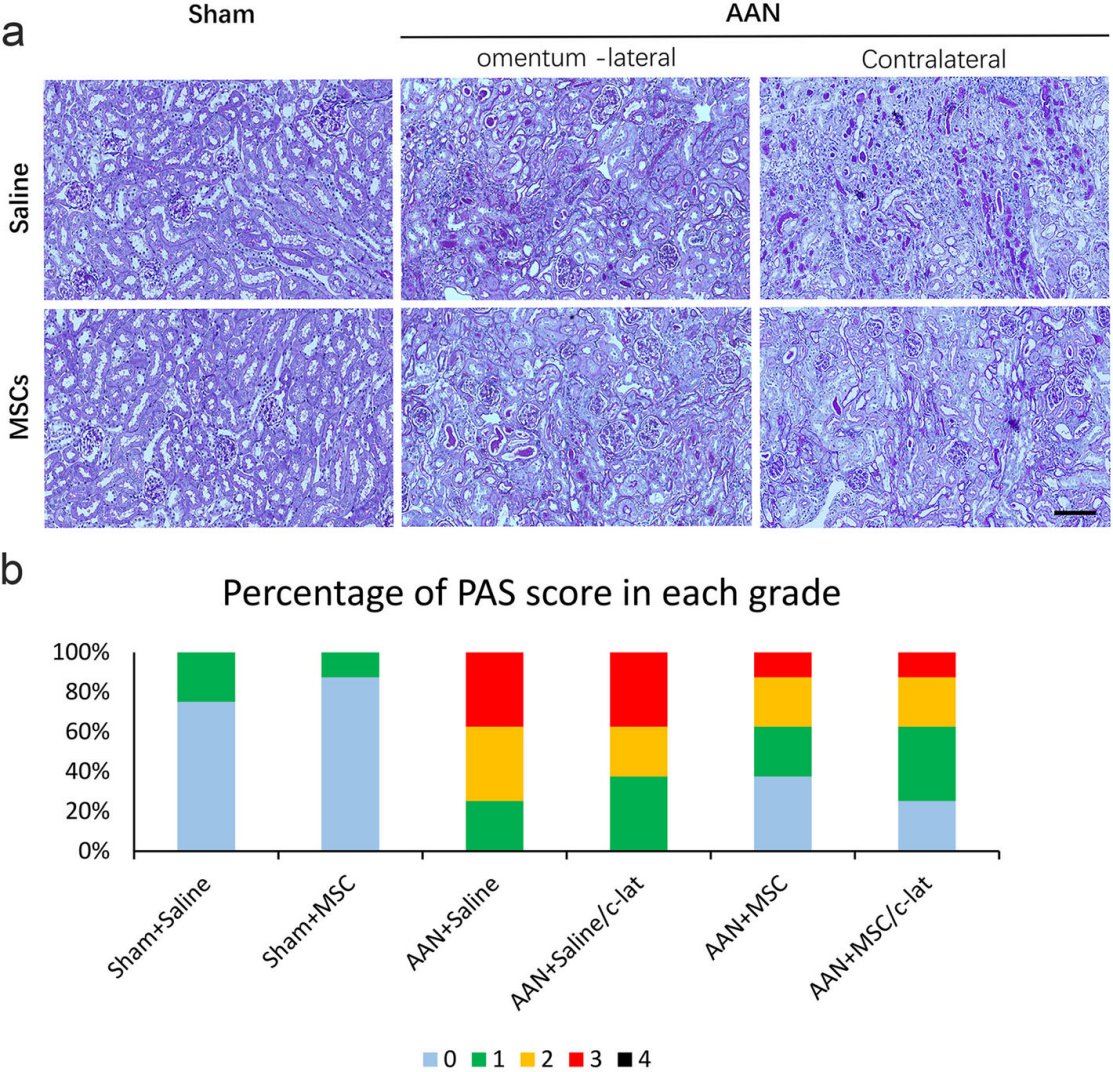
MSCs ameliorate renal injury induced by AA-I. **a** PAS staining of the kidney showing different grades of tubular injury at week 8 in sham+saline, sham+MSCs, AAN + saline, AAN + saline/c-lat, AAN + MSCs, and AAN + MSCs/c-lat mice. After the MSC treatment, the area of deposition in AAN-MSC mice was significantly smaller than that in AAN-saline mice. **b** Bar graphs showing the percentage of PAS score in each grade. A higher percentage of higher scores was observed in the AAN-saline group than in the AAN-MSC group. A significant difference was not observed between the omentum-wrapped kidney (the left kidney) and contralateral kidney (the right kidney). Score of 0: < 1%; score of 1: 1–5%; score of 2: 6–10%; score of 3: 11–20%; and score of 4: 21–50%. Data are presented as the means ± SEM (*n* = 8). Scale bar = 400 μm

The severity of kidney injury was evaluated to examine the therapeutic effects of MSCs on week 8 by performing PAS staining of the injured kidney. Tissue sections from the sham groups showed normal glomeruli and tubules, but a disorganized tubular structure, tubular necrosis, tubular dilatation, and protein aggregate formation were observed in the AAN groups (Fig. 7). A large number of renal tubular epithelial cells showed cloudy swelling, granular degeneration, disintegration, detachment, and basement membrane exposure. Perivascular and interstitial fibrosis and inflammatory cells were also observed. Glomerular and tubular injury-related changes were observed in the AAN-saline group compared with the sham group. Although the sample size was limited, the difference in the PAS staining score was not statistically significant (*P* = 0.073). Injury and fibrosis were significantly reduced in the AAN-MSC group compared with the AAN-saline group, despite the degeneration and necrosis of tubular epithelial cells observed using PAS staining. Bar graphs also showed a higher percentage of mice with a high score in the AAN-saline group than in the AAN-MSC group. No significant difference was observed between the omentum-wrapped kidney (the left kidney) and the contralateral kidney (the right kidney).

**Fig. 8:**
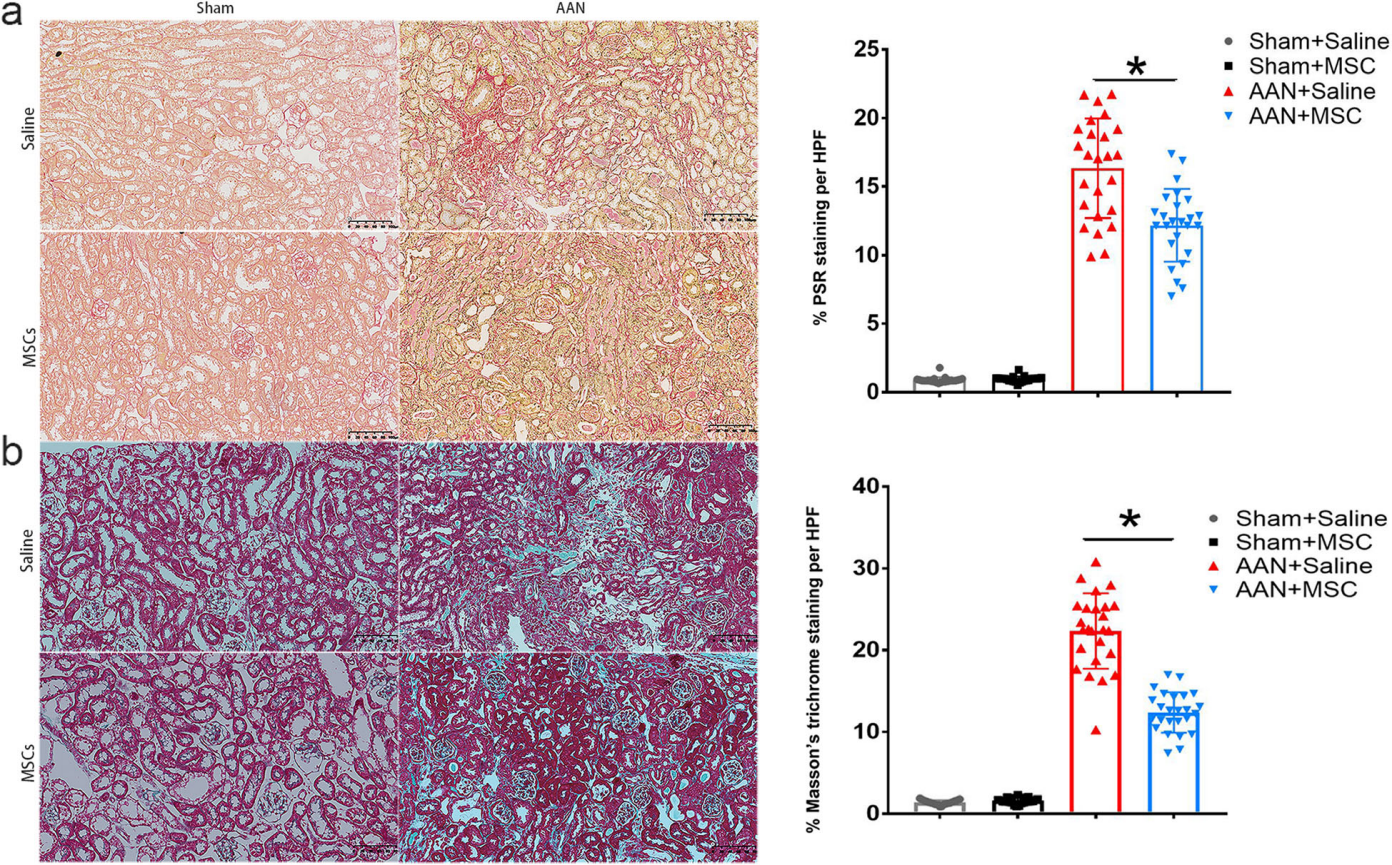
MSCs ameliorate renal injury induced by AA-I. Images of Sirius red staining (**a**) and Masson’s trichrome staining (**b**) show different grades of tubular injury at week 8 in sham+saline, Sham+MSCs, AAN + saline, and AAN + MSCs mice. After the MSC treatment, the area of deposition in AAN-MSC mice was significantly smaller than that in AAN-saline mice. Data are presented as the means ± SEM (*n* = 8). Scale bar = 400 μm. *P < 0.05 for the comparison between AAN-saline and AAN-MSC mice

We next performed Sirius red staining and Masson’s trichrome staining to evaluate tissue fibrosis. Sirius red staining and Masson’s trichrome staining revealed that AA-I induced collagen deposition in both AAN-saline and AAN-MSC mice, but the area of deposition in the AAN-MSC mice was significantly smaller than that in AAN-saline mice (*P* < 0.05) (Fig. 8). We concluded that ultrasound-guided administration of MSCs combined with greater omentum accelerated the recovery of renal function.

### MSCs alleviate fibrosis induced by AA-I

**Fig. 9:**
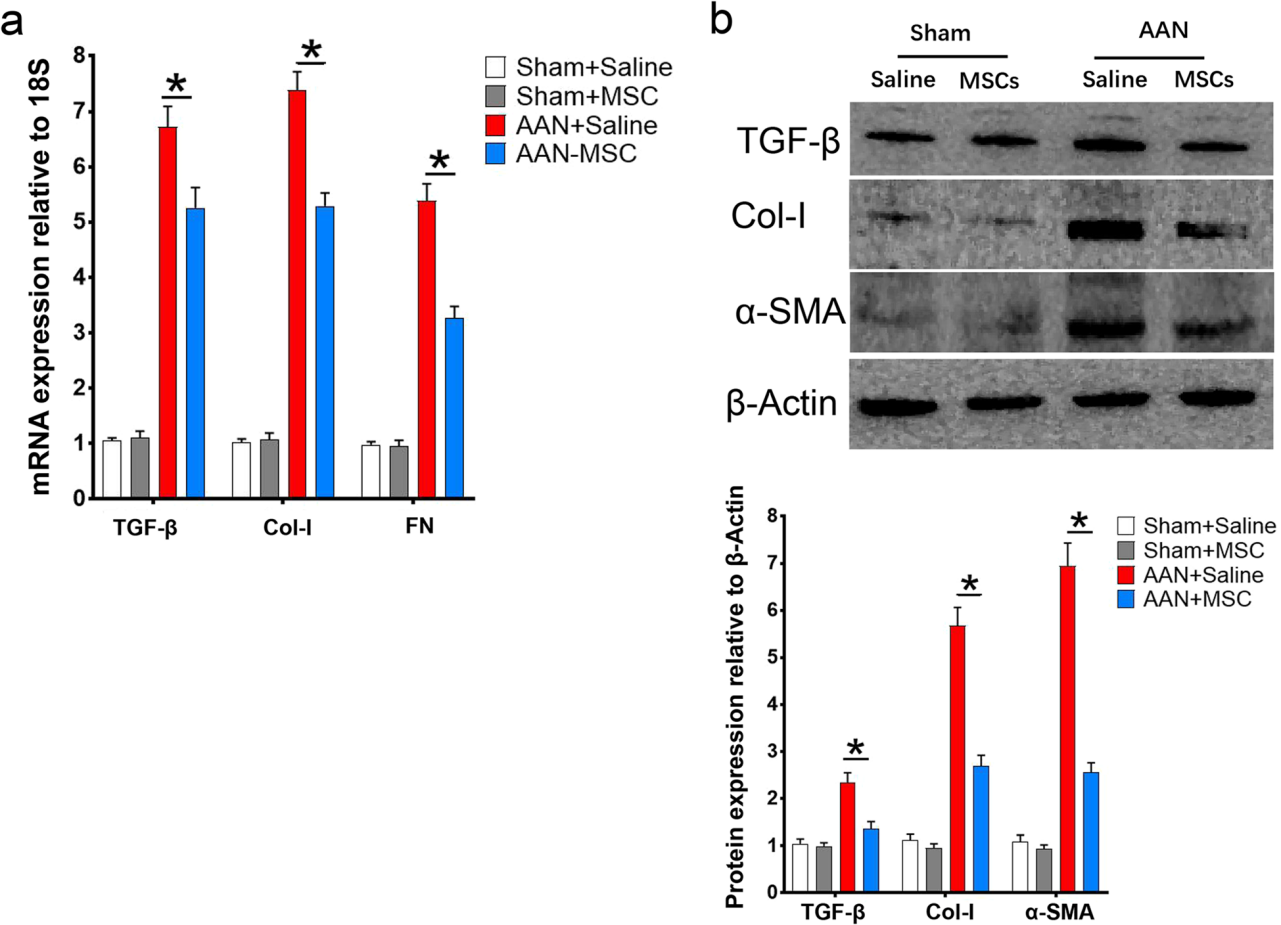
MSCs ameliorated kidney fibrosis in mice. **a** The mRNA expression levels of the fibrosis-related genes TGF-β, Col-I, and FN in the sham+saline, sham+MSC, AAN-saline, and AAN-MSC groups detected using qPCR. **b** Western blots showing the levels of TGF-β, Col-I, and α-SMA in the kidneys from the sham+saline, sham+MSC, AAN-saline, and AAN-MSC groups. The quantitative analysis of mRNA expression showed lower expression of TGF-β, Col-I, and FN in the AAN-MSC group than in the AAN-saline group. The quantitative analysis of protein expression showed lower expression of TGF-β, α-SMA, and Col-I in the AAN-MSC group than in the AAN-saline group. Data are presented as the mean ± SEM (*n* = 8). *P < 0.05 for the comparison between AAN-saline and AAN-MSC mice

As Col-I and fibronectin (FN) are the most critical ECM constituents in renal fibrosis, we first evaluated dynamic changes in the expression of these genes during kidney fibrosis by performing a qPCR analysis. The expression of Col-I and FN was upregulated in the AAN groups (AAN-saline and AAN-MSCs) compared with the sham groups (sham-saline and sham-MSCs). Following treatment with MSCs, the upregulation of fibrosis-related genes was significantly attenuated in the AAN-MSC group compared with the AAN-saline group (*P* < 0.05) (Fig. 9a). TGF-β/Smad signaling plays an important role in mediating renal fibrosis. We therefore measured whether increased Col-I and FN mRNA expression correlated with expected changes in TGF-β mRNA expression. Consistent with these findings, the expression of the TGF-β mRNA was significantly increased in the kidney tissues from the AAN groups (AAN-saline and AAN-MSCs) compared with the sham groups (sham-saline and sham-MSCs). After treatment with MSCs, a reduction in TGF-β expression was observed.

The levels of the TGF-β, Col-I, and α-SMA proteins were subsequently evaluated. Western blot analyses revealed increased expression of TGF-β, α-SMA, and Col-I in the AAN groups compared with the sham groups. Compared with the AAN-saline group, the AAN-MSC group showed a marked reduction in the levels of TGF-β, α-SMA, and Col-I in the kidney tissue (*P* < 0.05) (Fig. 9b).

**Fig. 10:**
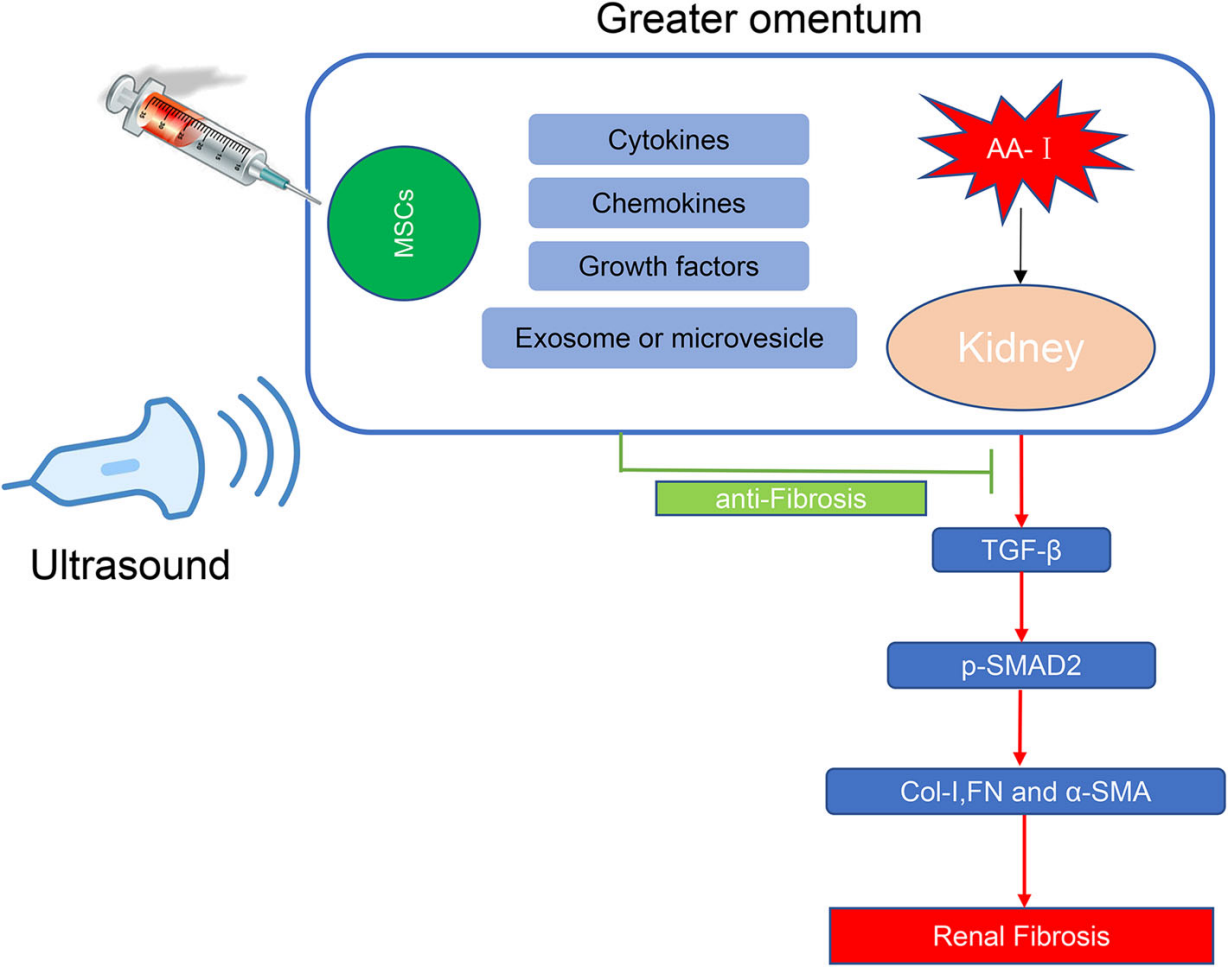
Schematic illustration of the involvement of the TGF-β/Smad signaling pathway in the therapeutic anti-fibrotic mechanisms of MSCs. MSCs downregulated the expression of Col-I, FN, and α-SMA by reducing TGF-β and p-Smad2 levels through the secretion of a wide variety of cytokines, chemokines, growth factors, and exosomes or microvesicles

**Fig. 11:**
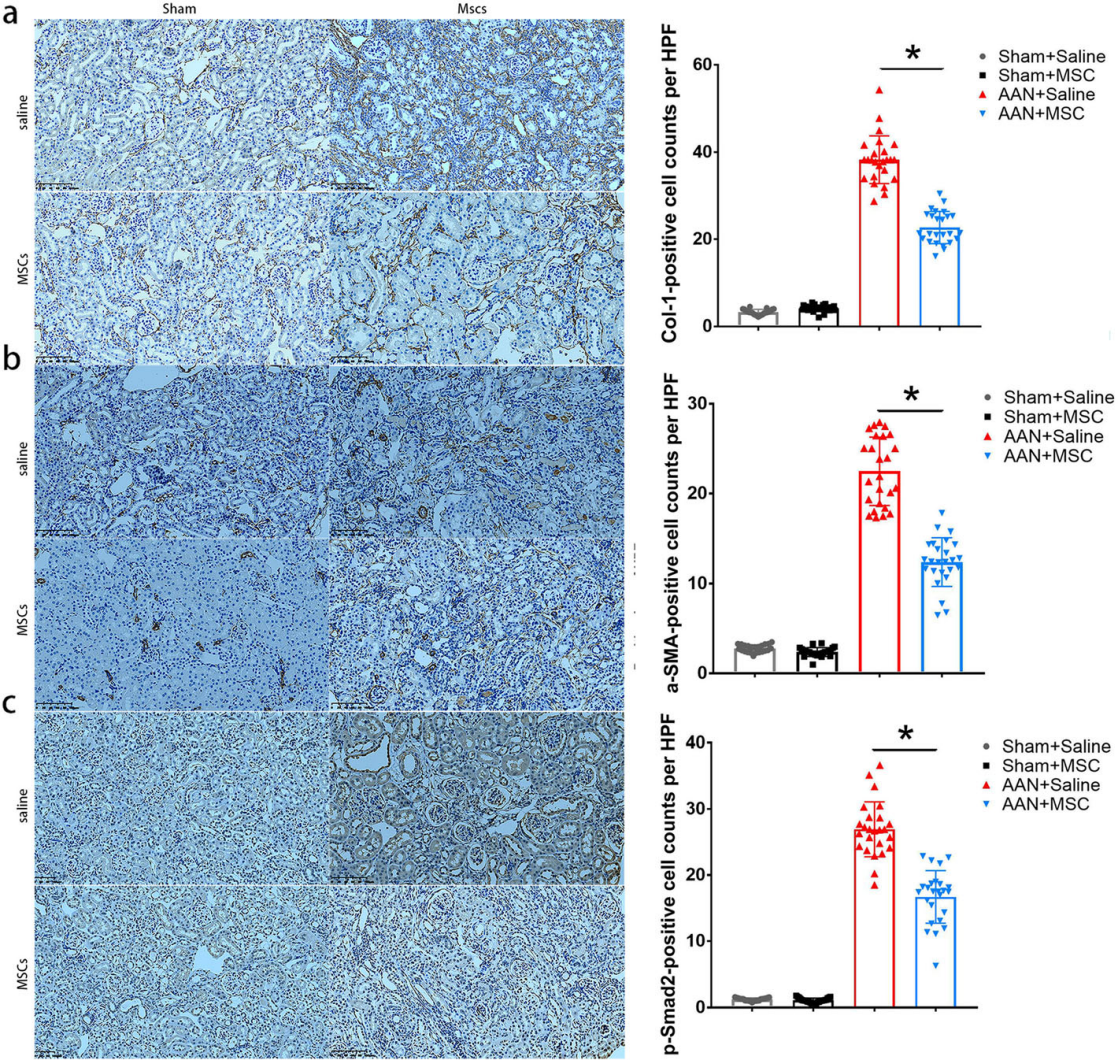
Antifibrotic effect of MSCs at 8 weeks after inducing AAN in mice. **a** Immunohistochemical staining for Col-1, **b** α-SMA, and **c** p-SMAD2 in kidney tissues from the sham+saline, sham+MSC, AAN-saline, and AAN-MSC groups. The magnification for Col-1, α-SMA, and p-SMAD2 staining was 400X (scale bar, 400 μm). Immunohistochemical staining confirmed the downregulation of fibrosis indexes in the AAN-MSC group compared with the AAN-saline group. Data are presented as the means ± SEM (*n* = 8). *P < 0.05 for the comparison between AAN-saline and AAN-MSC mice

Finally, we verified that the TGF-β/Smad signaling pathway was involved in this process by performing immunohistochemical staining. MSCs secrete a wide variety of cytokines, chemokines, growth factors, and exosome or microvesicles that contribute to decrease the levels of TGF-β and p-Smad2, and hence downregulated the expression of Col-I, FN, and α-SMA (Fig. 10). Immunohistochemical staining indicated very low constitutive staining for Col-I, α-SMA, and p-SMAD2 in sham-operated animals (Fig. 11). In contrast, samples obtained from AAN mice showed increased Col-I, α-SMA, and p-SMAD2 staining. The fibrosis indexes in the AAN-MSC group were lower than those in the AAN-saline group (*P* < 0.05).

### MSC exerts an anti-inflammatory effect

**Fig. 12:**
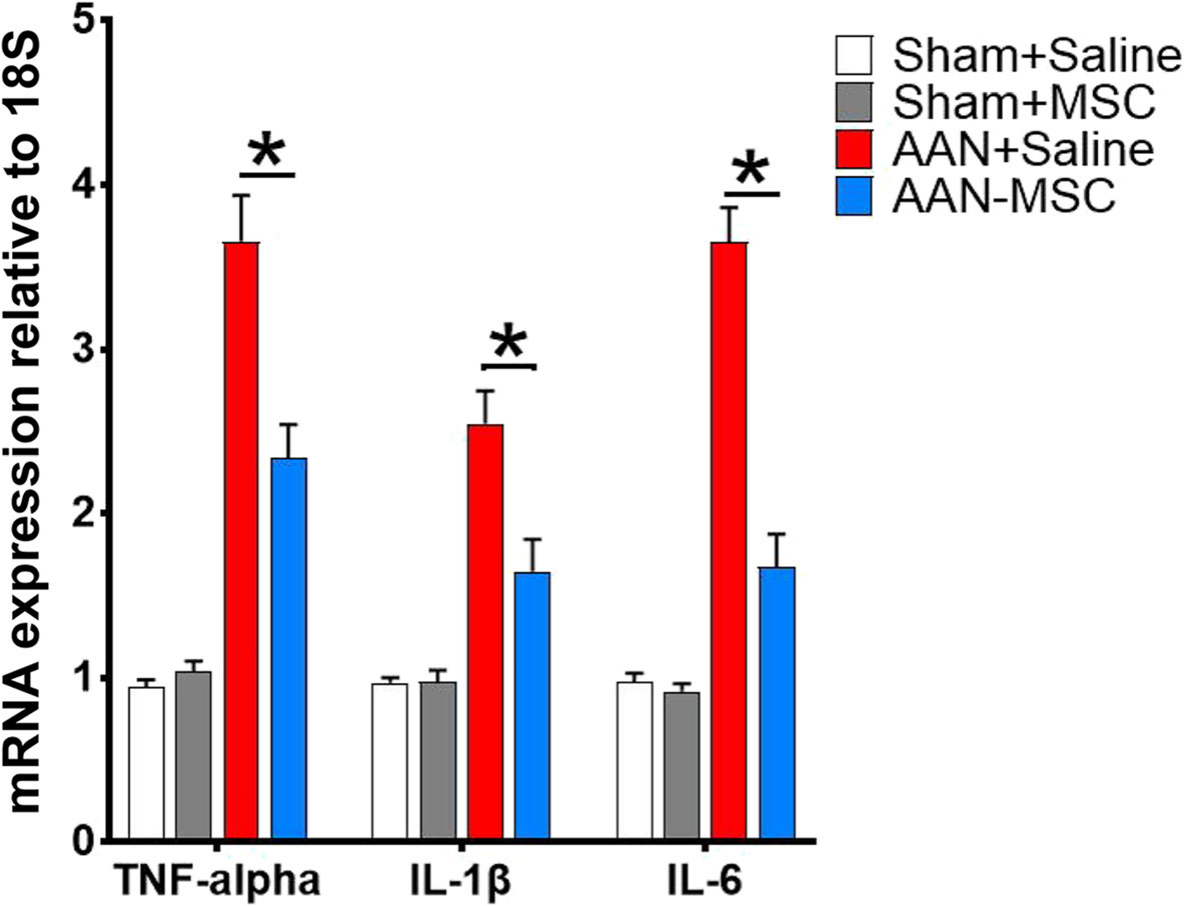
MSCs ameliorated the kidney inflammatory response in mice. The mRNA expression levels of the inflammatory-related genes TNF-alpha, IL-1β, and IL-6 in the sham+saline, sham+MSC, AAN-saline, and AAN-MSC groups were detected using qPCR analysis. qPCR analysis confirmed the downregulation of the inflammatory genes TNF-alpha, IL-1β, and IL-6 in the AAN-MSC group compared with the AAN-saline group. Data are presented as the means ± SEM (*n* = 8). *P < 0.05 for the comparison between AAN-saline and AAN-MSC mice

Inflammation plays a critical role in the progression of renal fibrosis. We suspected that a subsequent change in the strength of the inflammatory response occurred. The expression of inflammatory factors, including tumor necrosis factor-alpha (TNF-alpha), interleukin-1β (IL-1β), and interleukin-6 (IL-6), was also assessed in all animals using real-time RT-PCR (Fig. 12). The inflammatory cytokines TNF-alpha, IL-1β, and IL-6 were upregulated in the AAN groups (AAN-saline and AAN-MSC) compared with the sham groups (sham-saline and sham-MSC) (*P* < 0.05). In addition, this effect was dramatically mitigated by MSCs in the AAN-MSC group compared with the AAN-saline group (*P* < 0.05).

**Fig. 13:**
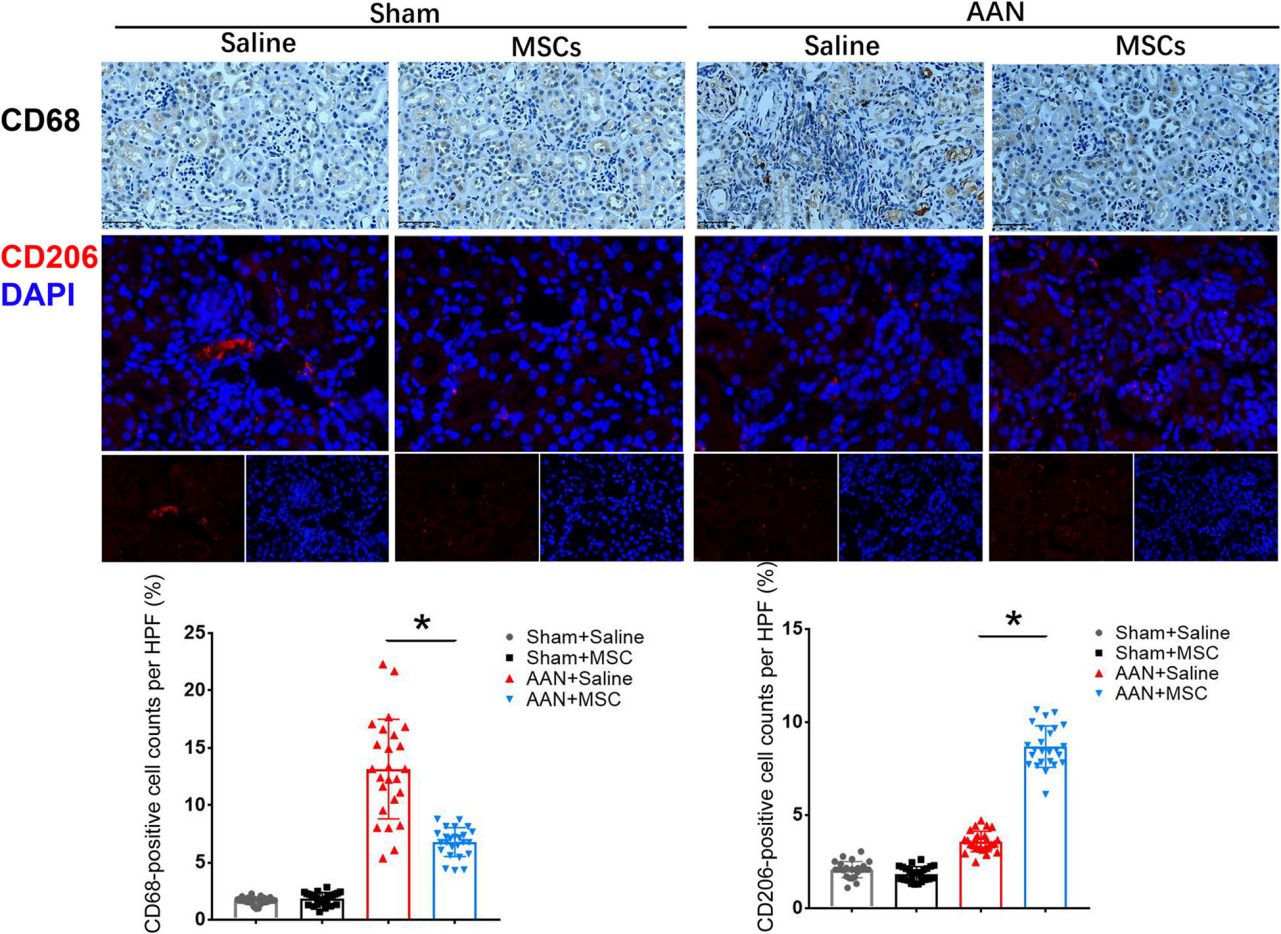
Anti-inflammatory effect of MSCs at 8 weeks after AAN induction in mice. Immunofluorescence staining for CD68 and CD206 in kidney tissues from the sham+saline, sham+MSC, AAN-saline, and AAN-MSC groups. The magnification for CD68 and CD206 staining was 400X (scale bar, 400 μm). Immunohistochemical staining confirmed the decrease in CD68+ cell numbers and the increase in CD206+ cell numbers in the AAN-MSC group compared with the AAN-saline group. Data are presented as the means ± SEM (*n* = 8). *P < 0.05 for the comparison between AAN-saline and AAN-MSC mice

We examined general macrophage markers using immunohistochemistry for CD68 and detected M2 macrophage markers using immunofluorescence labeling for CD206 to further investigate the effect of MSCs on inflammation (Fig. 13). Numbers of both CD68+ and CD206+ cells were increased in the AAN groups (AAN-saline and AAN-MSC) compared with those in the sham groups (sham-saline and sham-MSC), and the AAN-MSC group showed a significant decrease in CD68+ cell numbers but a significant increase in CD206+ cell numbers compared with those in the AAN-saline group (*P* < 0.05).

## Discussion

Currently, two hypotheses about MSC treatment mechanisms are generally recognized. Some studies have shown that MSCs differentiate into renal cells [22–24], which repair renal injury. However, in recent years, an increasing number of studies have suggested that the renoprotective effects of MSCs are mainly attributed to paracrine mechanisms [8, 25, 26]. Our findings support the latter hypothesis mainly for two reasons. Initially, MSCs did not enter the renal parenchyma, as detected using two-photon laser scanning microscopy. Second, fluorescence imaging showed that the fluorescence mainly accumulated in the left kidney region, and no fluorescence was detected in the right kidney region, while PAS staining showed no significant difference between the omentum-wrapped kidney and contralateral kidney. The Transwell migration assay suggested that both AA-I and TNF-α caused MSC migrate to the injured renal epithelial cells in vitro. The stronger migration-promoting ability of TNF-α is most likely attributed to its high concentration that exceeds the concentration in the cellular microenvironment. We inferred that MSCs attached to the surface of the left kidney and the greater omentum due to the secretion of inflammatory factors, including TNF-α, induced by AA-I.

Various approaches have been developed to improve the efficacy of MSC-based therapeutics for CKD after AKI, including an incubation with cytokines or chemical compounds [27–30] and the application of supporting materials [19]. At the same time, different injection methods, including intravenous or arterial injection, might also affect the efficacy of MSCs. Intravenous or intra-arterial MSC injection is relatively simple, but an important problem is that the homing rate to the target organs is relatively low and very few stem cells arrive at and remain in the kidney [31, 32]. Although some studies claimed that intra-arterial injection prevents the initial uptake of MSCs by systemic organs, fewer MSCs (1–10%) arrive at the injured lesion even with intra-arterial injection is performed [33]. In addition to these methods, other options should be developed to increase the efficacy of MSC therapies.

In our experimental design, MSCs were transplanted into the cavity formed by the left kidney and the greater omentum to ensure that MSCs were attached to the greater omentum or the surface of the kidney damaged by AA-I to exert paracrine effects. Although MSCs were maintained in the kidney region at least for 5 days, we injected MSCs into the greater omentum on the renal surface under B-ultrasound guidance once a week to achieve stable and continuous treatment and further ensure the curative effect.

Our results revealed that MSCs promote AA-I-induced kidney injury and renal functional recovery by inhibiting the renal interstitial fibrosis pathway and promoting anti-inflammatory responses, as shown by their effects on TGF-β, α-SMA, p-SMAD2, Col-I, TNF-alpha, IL-1β, and IL-6 expression and kidney histology. TGF-β is an important regulatory factor in the TGF-β/SMAD pathway. It increases the synthesis of ECM proteins, including Col-I and FN, and the expression of TGF-β may reflect renal fibrosis [34]. α-SMA is a marker of activated MyoF that is closely related to the proliferation of mesangial cells. The expression of α-SMA in the kidney indirectly reflects the number of MyoF, and thus, α-SMA is widely used as an index to detect the phenotypic transformation of renal intrinsic cells into MyoF [35]. In our investigation, the production of TGF-β, α-SMA, Col-I, and p-SMAD2 was reduced in the AAN-MSC group compared with the AAN-saline group, suggesting that MSCs show antifibrotic activity.

Compared with the AAN-saline group, the expression of the inflammatory factors TNF-alpha, IL-1β, and IL-6 decreased in the AAN-MSC group, indicating that MSCs showed anti-inflammatory activity. These results are consistent with our previous study showing that the application of pedicled greater omentum flaps loaded with MSC-loaded gelatine microcryogels for the treatment of 5/6 nephrectomized kidneys significantly improved the function of the residual kidney via anti-fibrotic and anti-inflammatory mechanisms [19]. When the expression of TNF-α, a proinflammatory cytokine, was downregulated, CD206 expression in the AAN-MSC group was increased compared with that in the AAN-saline group. The finding is consistent with a previous report by Li et al. of an AKI animal model showing that depletion of M2 macrophages leads to more severe kidney damage and worse renal function because M2 macrophage depletion increases TNF-α secretion during sepsis-induced AKI [36]. At the same time, the expression of the pro-inflammatory signaling molecule CD68 exhibited a statistically significant decrease in the AAN-MSC group. Taken together, we concluded that MSCs exert an anti-inflammatory effect by attenuating M1 activation and promoting M2 polarization.

Based on our results, wrapping MSCs with greater omentum is a good approach to enrich MSCs and increase the survival rate of MSCs. First, the greater omentum has specific features and advantages compared with intravenous or intra-arterial injection. When the greater omentum comes into contact with other tissues, capillaries begin to grow for approximately 6 h, and thus, the greater omentum can provide good conditions for the survival of MSCs [37]. In our study, MSCs wrapped in pedicled greater omentum flaps were maintained in the kidney region for at least 5 days. Second, the omentum functioned to physically confine the MSCs and enhanced the local paracrine signaling in an animal model of AAN [38]. Third, it avoided the first-pass effect by systemic organs. Fourth, mice were able to receive high-dose MSCs (2 × 10^7^) at one time. Fifth, the administration of multiple doses can be performed with the aid of ultrasound to maintain efficacy and compensate for the apoptosis of MSCs after exposure to uremic toxins during CKD. Last but not least, ultrasound is a valuable tool that will allow us to pinpoint the location of the cavity without mechanical complications.

Of course, our experimental approach also has drawbacks. The harvested kidneys should have been imaged using small animal live imaging ex vivo to verify whether MSCs attached to the surface of the left kidney and the greater omentum. Follow-up studies will continue to address this issue.

## Conclusions

In summary, we propose that under ultrasound guidance, treatment with MSCs wrapped in greater omentum exerts a renoprotective effect on AA-I-induced kidney injury in mice. On the one hand, MSCs wrapped in greater omentum directly contact the targeted left kidney, which solves the problem of stem cell homing. However, MSCs wrapped in greater omentum do not enter the renal parenchyma. On the other hand, repeated injections may ensure a sustained and stable therapeutic effect. This treatment can be used as an auxiliary method of stem cell therapy, which may provide new ideas for combination therapies for the treatment of AAN in the future.

## Supplementary Information

**Additional file 1.** Language editing certificate.

## Data Availability

All data generated or analyzed during this study are available from the authors.

## Abbreviations

MSCs: Mesenchymal stem cells
AKI: Acute kidney injury
CKD: Chronic kidney disease
AAN: Aristolochic acid nephropathy
RFP-MSCs: Red fluorescent protein-mesenchymal stem cells
AA-I: Aristolochic acid I sodium salt
RMV: Real-time microvisualization
RT-PCR: Real-time polymerase chain reaction
FN: Fibronectin
RTEC: Renal tubular epithelial cell
MyoF: Myofibroblasts
TGF-β: Transforming growth factor-β
α-SMA: α-Smooth muscle actin
p-SMAD2: Phosphorylated Smad2
Col-I: Collagen I
