# Supplementary material for: Ultrasound enhances the therapeutic potential of mesenchymal stem cells wrapped in greater omentum for aristolochic acid nephropathy
Source: Stem Cell Res Ther. 2021 May 3;12:261. doi: 10.1186/s13287-021-02243-7 (PMC8091698; doi:10.1186/s13287-021-02243-7)
Supplement: Supplementary file 1 — Additional file 1. Language editing certificate. [file 13287_2021_2243_MOESM1_ESM.pdf]

# Editing Certificate

This document certifies that the manuscript

## Ultrasound enhances therapeutic potential of mesenchymal stem cells wrapped in greater omentum for AAN

prepared by the authors

**Yuanjun Yang†, Xiaodong Geng†, Kun Chi, Chao Liu, Ran Liu, Xiangmei Chen, Quan Hong* and Guang-Yan Cai***

was edited for proper English language, grammar, punctuation, spelling, and overall style by one or more of the highly qualified native English speaking editors at AJE.

This certificate was issued on **February 15, 2021** and may be verified on the AJE website using the verification code **71C7-EB81-6098-59C0-A4CP** .

Neither the research content nor the authors' intentions were altered in any way during the editing process. Documents receiving this certification should be English-ready for publication; however, the author has the ability to accept or reject our suggestions and changes. To verify the final AJE edited version, please visit our verification page at aje.com/certificate. If you have any questions or concerns about this edited document, please contact AJE at support@aje.com.

AJE provides a range of editing, translation, and manuscript services for researchers and publishers around the world. For more information about our company, services, and partner discounts, please visit aje.com.
